# Reconstruction of the Global Polarity of an Early Spider Embryo by Single-Cell and Single-Nucleus Transcriptome Analysis

**DOI:** 10.3389/fcell.2022.933220

**Published:** 2022-07-22

**Authors:** Yasuko Akiyama-Oda, Takanori Akaiwa, Hiroki Oda

**Affiliations:** ^1^ JT Biohistory Research Hall, Takatsuki, Japan; ^2^ PRESTO, Japan Science and Technology Agency, Kawaguchi, Japan; ^3^ Department of Microbiology and Infection Control, Faculty of Medicine, Osaka Medical and Pharmaceutical University, Takatsuki, Japan; ^4^ Department of Biological Science, Graduate School of Science, Osaka University, Toyonaka, Japan

**Keywords:** spider, arthropod, pattern formation, transcriptome, single-nuclei RNA-seq (snRNA-seq), single-cell RNA-seq (scRNA-seq), embryo, axis formation

## Abstract

Patterning along an axis of polarity is a fundamental step in the development of a multicellular animal embryo. In the cellular field of an early spider embryo, Hedgehog signaling operates to specify a “fuzzy” French-flag-like pattern along the primary axis, which is related to the future anterior–posterior (A–P) axis. However, details regarding the generation and development of a diversity of cell states based on the embryo polarity are not known. To address this issue, we applied single-cell RNA sequencing to the early spider embryo consisting of approximately 2,000 cells. Our results confirmed that this technique successfully detected 3 cell populations corresponding to the germ layers and some transient cell states. We showed that the data from dissociated cells had sufficient information for reconstruction of a correct global A–P polarity of the presumptive ectoderm, without clear segregation of specific cell states. This outcome is explained by the varied but differentially overlapping expression of Hedgehog-signal target genes and newly identified marker genes. We also showed that the data resources generated by the transcriptome analysis are applicable to a genome-wide search for genes whose expression is spatially regulated, based on the detection of pattern similarity. Furthermore, we performed single-nucleus RNA sequencing, which was more powerful in detecting emerging cell states. The single-cell and single-nucleus transcriptome techniques will help investigate the pattern-forming processes in the spider model system in an unbiased, comprehensive manner. We provided web-based resources of these transcriptome datasets for future studies of pattern formation and cell differentiation.

## Introduction

In the cellular field of a developing tissue or embryo, a reproducible complex pattern of gene expression forms through dynamic gene regulations and cell–cell interactions. Formation of spatial periodic stripe patterns in the *Drosophila* blastoderm embryo has long stood as a simple, excellent model system to study and understand pattern-forming processes and the underlying mechanisms in multicellular animals (Bate and Arias, 1993). However, most of the patterning process in this *Drosophila* system occurs in a syncytial environment, allowing transcription factors and cytoplasmic components to diffuse over a long range (Driever and Nüsslein-Volhard, 1988a). This peculiar situation may limit applicability and generalization of our knowledge from the *Drosophila* patterning system.

The spider *Parasteatoda tepidariorum*, which together with *Drosophila* belongs to the phylum Arthropoda, has emerged as a model organism to study pattern-forming processes and the underlying mechanisms in a cellular field (Hilbrant et al., 2012; Oda and Akiyama-Oda, 2020). In this spider embryo, a segmented germ band with conserved stripes of gene expression forms like *Drosophila*; however, the cellular and molecular processes leading to these similar outcomes are highly different. In the early *Parasteatoda* embryo, cellularization is achieved by the stage when the number of the cleavage nuclei increases to sixteen (Kanayama et al., 2010), and the A-P and dorsal-ventral (D-V) axes and periodic segmental stripes are specified by cell–cell interactions mediated by signaling pathways (Akiyama-Oda and Oda, 2003, Akiyama-Oda and Oda, 2006, Akiyama-Oda and Oda, 2010, Akiyama-Oda and Oda, 2020; McGregor et al., 2008; Kanayama et al., 2011; Schönauer et al., 2016; Setton and Sharma, 2021). The Hedgehog (Hh) signaling pathway regulates the formation of global polarity along the primary axis: an axis of radial symmetry that emerges during the formation of a disc-shaped epithelial cell sheet called the germ disc. The roles of Hh signaling pathway in the early *Parasteatoda* embryo are similar to those of maternal transcription factor morphogens, such as Bicoid, in the early *Drosophila* embryo (Driever and Nüsslein-Volhard, 1988b; Briscoe and Small, 2015). Hh signaling plays key roles in a variety of tissue patterning events in bilaterians, including D-V patterning in the vertebrate neural tube (Dessaud et al., 2008; Ribes and Briscoe, 2009; Placzek and Briscoe, 2018), digit patterning in the vertebrate appendage primordia (Tickle and Towers, 2017; Zhu and Mackem, 2017), and the generation of repetitive ridges in the mammalian palate (Economou et al., 2012). In these patterning tissue fields, Hh signaling network, owing to its positive and negative feedback loops (Briscoe and Thérond, 2013; Kong et al., 2019), dynamically controls the expression of genes in respective cells. However, these fields are located inside an embryo or organ and organized by dynamic cell populations, in which the position of individual cells is not easily followable. In contrast, the early *Parasteatoda* embryo, like the *Drosophila* blastoderm embryo, provides a simple, easily observable cellular field (but not syncytial), in which periodic stripe formation progresses (Hemmi et al., 2018) by the mechanisms mediated by Hh and other signaling pathways (McGregor et al., 2008; Kanayama et al., 2011; Schönauer et al., 2016; Akiyama-Oda and Oda, 2020; Setton and Sharma, 2021).

**FIGURE 1:**
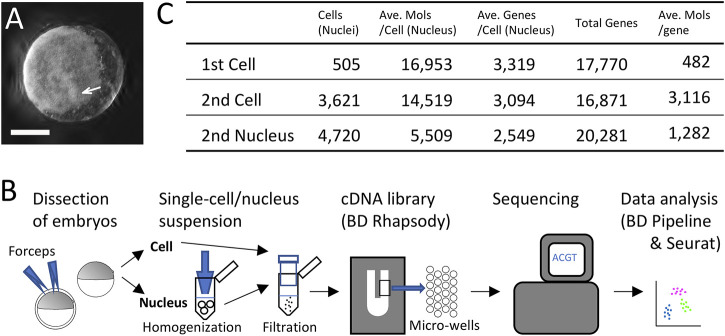
Single-cell and single-nucleus transcriptomics of the early spider embryo. **(A)** An embryo at late germ-disc stage (late stage 5). The germ disc is observed as the white sheet. The arrow indicates the cumulus. Scale Bar = 200 μm. **(B)** Diagram showing single-cell and single-nucleus experimental procedure. **(C)** Metrics summary after filtering out low-quality cells (nuclei). Numbers of cells and nuclei examined in the first single-cell, the second single-cell, and the single-nucleus analyses, average of detected molecules per cell (nucleus), average of detected genes per cell (nucleus), total numbers of detected genes, and average of detected molecules per gene.

The *Parasteatoda* late stage-5 germ disc (Figure 1A) is a large field of single-layered epithelial cell sheet that consists of approximately 2,000 morphologically uniform, undifferentiated cells (Akiyama-Oda and Oda, 2020). This field has global polarities reflecting the future A-P axis of the embryo but has not gained spatially periodic patterns of gene expression. The center of the germ disc, also referred to as the embryonic pole, corresponds to the future posterior pole, whereas the peripheral region of the germ disc corresponds to the future anterior. Most of the area of the germ disc develops into the surface ectoderm maintaining its continuity. The other germ layers, the endoderm and mesoderm, develop from cells internalized at and near the center of the germ disc and the rim of the germ disc (Yamazaki et al., 2005; Oda et al., 2007). A previous study using the combination of RNA interference (RNAi) and RNA sequencing (RNA-seq) identified many genes expressed in concentric circle patterns on the germ disc under the control of Hh signaling (Akiyama-Oda and Oda, 2020). Expression of genes positively regulated by Hh signaling is detected in the peripheral region of the germ disc, and expression of those negatively regulated is detected in the central region. Some genes that require both positive and negative regulations are expressed in the intermediate region. These situations remind us of the French flag model (Wolpert, 1969; Briscoe and Small, 2015); however, the boundaries of individual gene expression domains are obscure or fuzzy, and expression of some genes is sparse or dynamic on the static germ disc. Following the germ-disc stage, the cellular field undergoes a planar remodeling to transform into a germ band, in which an increasing number of stripes arise through dynamic regulation of wave-like expression of genes (Kanayama et al., 2011; Schönauer et al., 2016; Hemmi et al., 2018; Akiyama-Oda and Oda, 2020). To date, several genes expressed in specific regions of the germ disc that play roles for specific steps in *Parasteatoda* segmentation have been identified. These genes include an *orthodenticle* homolog *Pt-otd*, an *odd-paired* homolog *Pt-opa*, a *distal-less* homolog *Pt-dll,* and a *muscle segment homeobox* (*msh*) homolog *Pt-msx1* (Pechmann et al., 2009, Pechmann et al., 2011; Kanayama et al., 2011; Akiyama-Oda and Oda, 2016, Akiyama-Oda and Oda, 2020). The roles for some of these in segmentation were unpredictable based on our knowledge from the *Drosophila* model system. For example, in *Drosophila*, *msh* functions under the specification of several muscle cells and neuroectodermal cells (Lord et al., 1995; Isshiki et al., 1997), whereas in *Parasteatoda*, *Pt-msx1* is essential for generating gene expression dynamics involved in segmentation. These previous findings strongly suggest the need for a genome-wide, unbiased approach that can capture spatially regulated gene expression to investigate the pattern-forming processes in the new spider model system.

To this end, the present study tested the applicability of single-cell RNA-seq (Jaitin et al., 2014; Macosko et al., 2015) to the *Parasteatoda* embryo at the late germ-disc stage. We showed that this technique worked effectively using only twenty or less embryos for each experiment. Analyses of quantitative datasets obtained from dissociated cells successfully separated 3 cell populations corresponding to the presumptive ectoderm, mesoderm, and endoderm and reconstructed a global A-P polarity in the ectodermal cell population. We also performed single-nucleus RNA-seq (Grindberg et al., 2013; Habib et al., 2016), which produced similar results to those of single-cell RNA-seq, but with higher sensitivity to dynamic cell states emerging from the center of the germ disc. The resulting resources enabled us to conduct a genome-wide search *in silico* for genes whose expression is spatially regulated in the germ disc. The single-cell and single-nucleus transcriptome techniques will help investigate the pattern-forming processes in the spider model system in an unbiased, comprehensive manner.

## Materials and Methods

### Spider

Animal experiments were conducted according to the protocol reviewed and approved by the Institutional Animal Care and Use Committee of JT Biohistory Research Hall (No. 2020-1). We used laboratory stocks of the spider *Parasteatoda tepidariorum* (syn. *Achaearanea tepidariorum*), which were maintained at 25°C with a 16 h light/8 h dark cycle. Developmental stages have been described previously (Akiyama-Oda and Oda, 2003, Akiyama-Oda and Oda, 2010). We monitored the development of the eggs and determined the beginning of stage 5, when internalization of the cumulus mesenchymal (CM) cells is completed. The image of the spider embryo shown in Figure 1A was captured using a zoom microscope Zeiss Axio Zoom.V16 equipped with a digital camera Zeiss Axiocam506 (Carl Zeiss). Z-series images (5 μm × 56) were processed using an ImageJ plugin (Extended Depth of Field).

### Isolation of Single Cell and Single Nucleus

The spider embryos were dechorionated using 100% commercial bleach, rinsed with distilled water, and transferred into CGBS-CMF [Chan and Gehring buffered saline (Chan and Gehring, 1971), Ca and Mg-free: 55 mM NaCl, 40 mM KCl, 10 mM Tricine, pH6.95]−0.01% (for cell) or 0.5% (for nucleus) BSA (Nacalai tesque, 01278-44) solution. In this solution, vitelline membrane was manually removed with forceps. To obtain dissociated cells, the devitellinized sample was filtered through a 40-μm cell strainer (pluriStrainer-Mini, 43-10040-40) and collected in a DNA LoBind Tube (Eppendorf 022431021). For preparation of nuclei, the devitellinized sample was centrifuged in a 1.5-ml tube and suspended in 400 μL homogenization buffer (McLaughlin et al., 2021) [250 mM Sucrose, 10 mM Tris (pH 8.0), 25 mM KCl, 5 mM MgCl_2_, 0.1% Triton-X100, 0.1 mM DTT, 100 u/mL RNase Inhibitor (SUPERaseIn RNase Inhibitor: Invitrogen, AM2694), Protease Inhibitor at 1:100 dilution (Nacalai tesque, 25955-11)], where the sample was homogenized with a loose plastic pestle (fisher scientific, 12-141-368) by 40 strokes. Next, the sample was centrifuged and resuspended with CGBS-CMF-0.5% BSA-100 u/mL RNase Inhibitor solution, followed by filtration through a 40-μm cell strainer. The centrifugation and resuspension steps (without the filtration step) were repeated twice. Four volumes of CellCover (Anacyte Laboratories, 800-050) were added to the cell and nucleus samples, which were then stored at 4°C. To proceed to the library construction step, stored samples were centrifuged and resuspended with the CGBS-CMF-0.025% Tween 20 (first single-cell library), CGBS-CMF-0.01% BSA (second single-cell library), or CGBS-CMF-0.01% BSA-100 u/mL RNase Inhibitor (single-nucleus library) solution. In these solutions, the samples were loaded onto BD Rhapsody cartridges (BD Biosciences).

For the first single-cell transcriptome library construction, we used ten late stage-5 eggs (approximately 8 h from the beginning of stage 5) derived from an egg sac. For the second single-cell and single-nucleus transcriptome library construction, we used 20 eggs each derived from another egg sac. To prepare the nuclei sample, eggs were dechorionated at the end of stage 5/the start of stage 6 (9–10 h from the beginning of stage 5), and for the second single-cell sample, eggs were dechorionated 1 h 20 min later, when preparation of the nuclei sample was completed.

### Construction of Libraries and RNA Sequencing

Single-cell RNA-seq libraries were constructed using a BD Rhapsody Targeted mRNA and AbSeq Amplification kit (BD Biosiences, 633774) or a BD Rhapsody WTA Amplification kit (BD Biosiences, 633801) according to the manufacturer’s instructions. To construct the single-nucleus RNA-seq library, a BD Rhapsody WTA Amplification kit was used with some modifications: RNase Inhibitor (SUPERaseIn RNase Inhibitor) was added at 100 u/mL to the sample buffer supplied in the kit, which was used for the bead wash steps, Proteinase K was added at 0.5 mg/ml to the Lysis buffer, and the lysis step was prolonged from 2 min to 5 min. Sequencing was performed using the HiSeqX Sequencing system (Illumina) with 150 paired-end reads, and 8 bp index reads were also performed for the second single-cell and single-nucleus libraries.

### Sequence Alignment and Count Matrix Generation

RNA-seq reads were processed using the BD Rhapsody WTA analysis pipeline on the Seven Bridges Genomics cloud platform (Tzani et al., 2021) with the default parameters. The R1 reads, possessing information of the cell barcode and the adjusted unique molecular index (UMI), were used to identify cells and molecules. The R2 reads, which were second strand cDNA sequences, were aligned using the STAR index created by STAR-2.5.2b (Dobin et al., 2013) against the *Parasteatoda tepidariorum* genome (Schwager et al., 2017), GCF_000365465.2_Ptep_2.0 (all libraries) and GCF_000365465.3_Ptep_3.0 (second single-cell and single-nucleus libraries). To count both exonic and intronic reads, an annotation file (a gtf file) accompanied with GCF_000365465.3_Ptep_3.0 was modified and used to align the second single-cell and the single-nucleus libraries. Integrating cellular and molecular information obtained from R1 reads and results of the alignment of R2 reads, a cell/nuc and gene count matrix for each sample was generated (GSE201705). Count metrics are summarized in Supplementary Tables S1, S7.

### Preprocessing and Clustering

Using the Seurat package (v.4.0.3) (Satija et al., 2015), the generated count matrices were filtered based on both the number of unique genes and the total number of RNA molecules or the total number of RNA molecules to exclude low-complex cells/nuclei and potential doublets; the thresholds were as follows: cells having 2,000 to 4,800 genes and having less than 30,000 molecules for the first single-cell library, 6,866 to 27,465 molecules (50%–200% of the mode) for the second single-cell library, and 2,667 to 10,670 molecules (50%–200% of the mode) for the single-nucleus library. Metrics of these quality-filtered counts are shown in Figure 1C. Log-normalization, identification of variable features, scaling, principal component (PC) analysis, dimensionality reduction, cell clustering, and detection of cluster marker genes were performed on these counts according to instructions of the Seurat package. In the clustering analysis, we tried multiple parameter settings to confirm that the cell populations were similarly detected. In the clustering analyses using the single-cell datasets, putative ectodermal cells were constantly separated into two large populations with various parameter settings; differentially expressed genes (DEGs) (Supplementary Tables S2, S8) were identified between the two ectodermal populations that were detected using the following parameters: 50 PCs and resolution 0.5 for the first single-cell analysis and 20 PCs and resolution 0.1 for the second single-cell analysis. These parameter values were optimized in each case, so that smaller subclusters were not generated within the ectodermal populations. The identified DEGs were excluded from the downstream analyses. The clustering output was visualized using a uniform manifold approximation and projection (UMAP) plot embedded in the Seurat package. UMAP plots showing expression of all identified genes can be viewed in a web database at https://www.brh2.jp and have been deposited, including the DEGs, in the Figshare repository (doi:10.6084/m9.figshare.c.5963571).

### Production of Composite Images of Two or Three UMAP Plots

UMAP plots generated in gray-black colors using Seurat were converted into gray-scale images using the ImageJ (FIJI) software (version 2.3.0/1.53f). Next, they were transformed into negative images and overlayed to produce composites in RGB colors using the ImageMagick convert and combine function.

### Identification of Cell Cycle Genes

*Parasteatoda* cell-cycle genes were searched using amino-acid sequences of *Drosophila* cell-cycle genes (https://github.com/hbc/tinyatlas/blob/master/cell_cycle/Drosophila_melanogaster.csv) as queries for tblastn searches against *Parasteatoda* genes, and nucleotide sequences of *Parasteatoda* top-hit genes were then used reciprocally for blastx searches against *Drosophila* genes. *Parasteatoda* putative orthologs of the *Drosophila* cell-cycle genes are listed in Supplementary Table S3.

### Construction of Dendrogram and Heatmap

We prepared three matrices of normalized counts from the first single-cell transcriptomes using 1) 411 cells belonging to clusters 2–5 (presumptive ectodermal cells), and the *Pt-hh* gene and 48 Hh-signaling target genes expressed in specific regions of the germ disc (total of 49 genes) (Akiyama-Oda and Oda, 2020); 2) 140 cells belonging to clusters 0–2 (presumptive endodermal, mesodermal, and peripheral ectodermal cells), and cluster 0 and cluster 1 markers and 20 peripheral genes that were selected from the above 49 genes (total of 137 genes); 3) 93 cells belonging to clusters 1 and 2 (presumptive mesodermal and peripheral ectodermal cells), and above 20 genes and markers of cluster 1 (total of 77 genes). Using R functions, Euclidian distances between cells were calculated and hierarchical clustering of cells was performed with the ward2 method. The results were visualized in dendrograms with the heatmap showing normalized counts.

### Identification of CM-Cell Markers

The expression of LOC107451717 (*Pt-Ets4*) (Pechmann et al., 2017) was used to identify a putative CM cell (#614005), and genes that showed specific expression at this cell were searched based on the following criteria: genes with normalized count >2 in the cell #614005 and genes whose expression was detected at < 20 cells.

### Image Similarity Search With Open CV Template Matching

A UMAP plot of LOC107444265 (*Pt-noggin-D*) (Akiyama-Oda and Oda, 2020) was used to identify genes specifically expressed at the cumulus. The area of the plot including *Pt-noggin-D*-positive and negative cells (Figure 6B’) was used as a template to search the corresponding areas of plots of all other genes for similar images. The background color of UMAP plots was set same as the color of the nonexpressing cells, and searches were performed using the python Open CV template matching module with the normalized correlation coefficient (CCOEFF_NORMED) method.

### cDNA Cloning

Full-length or partial cDNAs were obtained from our laboratory stocks of EST clones or were isolated using PCR. The resulting cDNAs were used to synthesize probes. The cDNA clones and the PCR primers used are listed in Supplementary Table S10.

### *In Situ* Hybridization and Image Acquisition

Whole mount *in situ* hybridization (WISH) and fluorescence *in situ* hybridization (FISH) were performed as described previously (Akiyama-Oda and Oda, 2003, Akiyama-Oda and Oda, 2016). Digoxigenin (DIG)-labeled probes were used for the alkaline-phosphatase chromogenic staining of WISH. Signals were amplified with a combination of anti-DIG-POD (used at 1:1000 dilution, Roche 11 207 733 910), dinitrophenyl (DNP)-tyramide (1:100 dilution, TSA plus DNP system, PerkinElmer), and anti-DNP-AP (1:100 dilution, Vector MB-3100) and were visualized with NBT/BCIP. In FISH, DIG and DNP probes were used in combination with anti-DIG-POD (used at 1:1000 dilution, Roche 11 207 733 910) and anti-DNP-HRP (used at 1:200 dilution, PerkinElmer FP1129) and 5-(and-6)-carboxyfluorescein and DyLight680 tyramides. Samples were counterstained with DAPI (Sigma-Aldrich) to visualize DNA.

Stained embryos were mounted on glass slides with spacers. WISH samples were observed using a stereomicroscope SZX12 (Olympus) equipped with a color 3CCD camera C7780-10 (Hamamatsu Photonics). FISH samples were observed using a TCS SPE confocal system (Leica). Images were processed using Imaris version 7.6.5 (Bitplane) and Adobe Photoshop CC 2020 and 2021 software.

## Results and Discussion

### Single-Cell Transcriptome and Clustering of Cells

In the first single-cell RNA-seq experiment, we first utilized dissociated cells from ten late germ-disc stage embryos (late stage 5) (Figure 1A). We constructed a single-cell cDNA library using the BD Rhapsody microwell-based platform, followed by sequencing on the Illumina platform and data processing using the BD Rhapsody WTA analysis pipeline (Supplementary Table S1) and the Seurat package v.4.0.3 (Figure 1B). Transcriptome data obtained from 505 single cells were used for subsequent analysis. On average, 16,953 molecules/cell transcribed from 3,319 genes/cell were detected with a total of 17,770 genes counted once or more in these 505 cells (Figure 1C).

Clustering analysis of these cells revealed 4 cell clusters (Supplementary Figure S1A). Expression of known markers indicated that two of the four clusters comprise germ-disc epithelial cells (presumptive ectoderm), with the other two comprising mesodermal and endodermal clusters (Supplementary Figure S1B) (Yamazaki et al., 2005; Akiyama-Oda and Oda, 2010). The two ectodermal clusters differed in the number of unique genes and molecules detected per cell (Supplementary Figure S1A) but similarly exhibited polarized expression of selected A-P marker genes (Akiyama-Oda and Oda, 2020) on the UMAP plot (Supplementary Figure S1B) (we will mention the polarization in detail below). To investigate the cause of the separation of two ectodermal cell populations, we identified 445 DEGs between the two ectodermal clusters (Supplementary Table S2). Expression of most of these DEGs was detected rather ubiquitously and at high levels (9,373 molecules/gene for the DEGs, in contrast to 482 molecules/gene for all counted genes on average, Figure 1C), although the expression levels were different between the two clusters (Supplementary Figure S1C, Supplementary Table S2). Biased levels of expression between the two clusters were not observed for cell-cycle genes except LOC107436263 (Supplementary Table S3, Supplementary Figure S1D). This indicated that differences in expression levels of highly expressed genes (the 445 DEGs), but not the cell-cycle states, were the main cause of the cluster separation.

**FIGURE 2:**
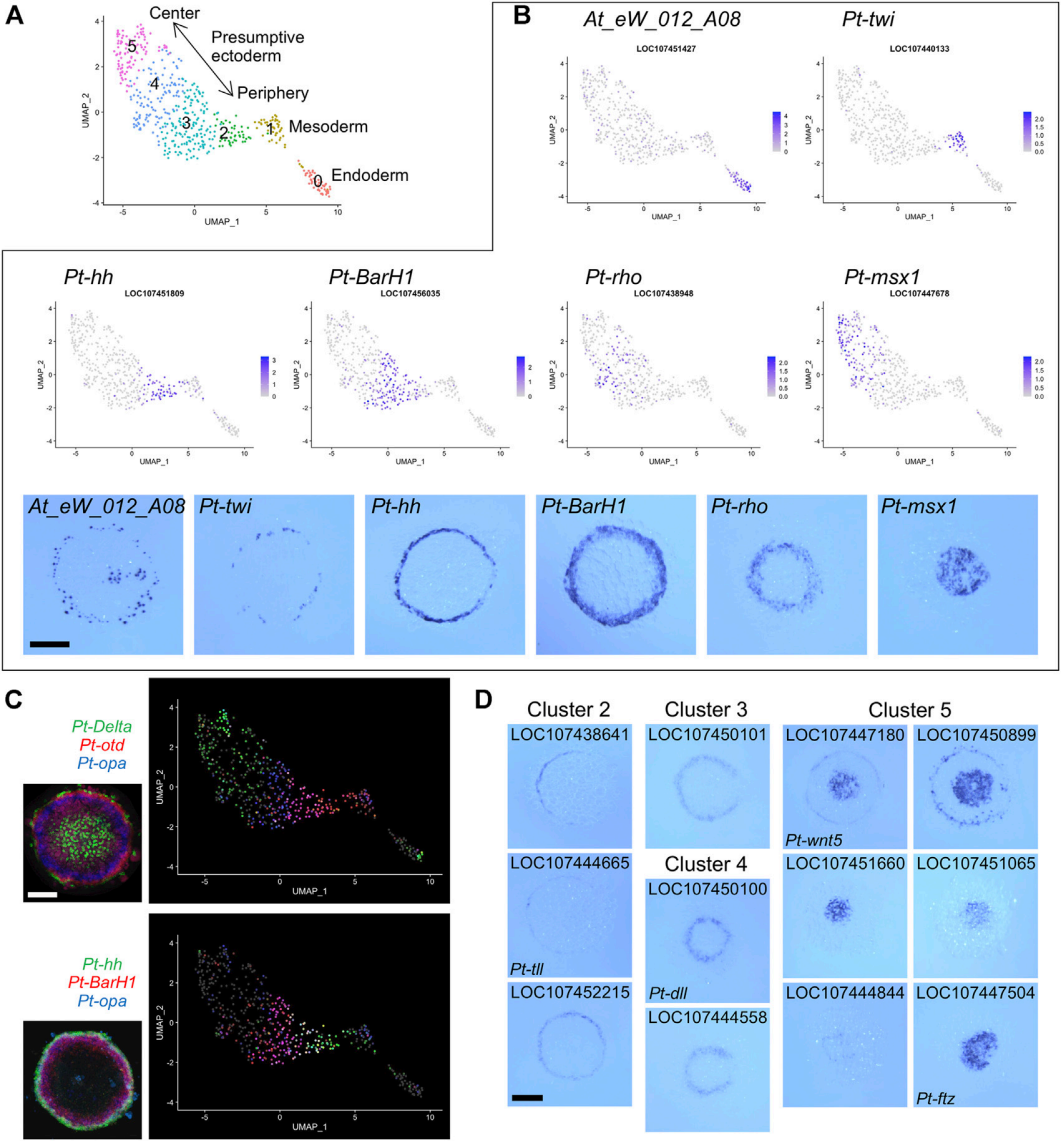
Detection of germ layers and the ectodermal polarity using single-cell transcriptomics. **(A)** Clustering analysis of the germ-disc stage embryonic cells. The UMAP plot of 505 cells shows presumptive endodermal (cluster 0), mesodermal (cluster 1), and ectodermal (clusters 2–5) clusters. The arrow shows the reconstructed orientation of the germ disc. The clustering was performed using 48 PCs with a resolution parameter of 1.0. **(B)** Visualization of the expression of germ-layer marker genes and A-P marker genes on the UMAP plot with the color code of the normalized expression levels and in the germ disc at late stage 5 using WISH. *At_eW_012_A08*, endoderm; *Pt-twi* (*twist*), mesoderm; *Pt-hh* (*hedgehog*), peripheral region; *Pt-BarH1*, broad peripheral region; *Pt-rho* (*rhomboid*), intermediate region; *Pt-msx1*, central region. **(C)** Visualization of the expression of three genes on the UMAP plot and in the late germ disc using multicolor FISH. *Pt-Delta* (green), *Pt-otd* (*orthodenticle*, red), and *Pt-opa* (*odd-paired*, blue); *Pt-hh* (green), *Pt-BarH1* (red), and *Pt-opa* (blue). The FISH images are from (Akiyama-Oda and Oda, 2020) (CCBY4.0). **(D)** Expression of marker genes identified for clusters 2–5 in the late germ disc. Scale Bars = 200 μm.

Re-clustering analysis following the exclusion of the 445 DEGs grouped cells into three populations, which corresponded to the presumptive endoderm, mesoderm, and ectoderm (Figures 2A,B). The first two populations (clusters 0 and 1, Figure 2A) were confirmed by expression of known markers, *At_eW_012_A08* (endoderm) (Akiyama-Oda and Oda, 2010) and *Pt-twist* (*Pt-twi*; mesoderm) (Yamazaki et al., 2005) (Figure 2B). New marker genes identified for these clusters were consistently expressed in the endoderm and mesoderm (Supplementary Table S4, Supplementary Figure S2). The endodermal markers identified by this single-cell transcriptome analysis of late stage 5 embryos largely overlapped genes identified by comparative transcriptome analyses using stage-3 and stage-5 embryos depleted of activity of a novel gata-family gene in another study (Iwasaki-Yokozawa et al., 2022). The two separate studies revealed a consistent endodermal gene set.

The presumptive ectodermal cells were subgrouped into four clusters (clusters 2-5, Figure 2A) without clear segregation of specific cell states and without clear differences in distributions of the numbers of molecules and genes per cell (Supplementary Figure S1E). Comparison of the expression patterns of selected A-P marker genes (Akiyama-Oda and Oda, 2020) visualized on the UMAP plot and detected on the germ disc using WISH indicated that the spatial order of the four clusters on the UMAP plot was correlated with the peripheral-to-central spatial order on the germ disc (Figure 2B). This correlation was further supported by multicolor visualization of expression of multiple A-P marker genes on the UMAP plot and the germ disc (Figure 2C). A similar correlation was observed even in a smaller spatial range of the UMAP plot and the germ disc (Figure 2C, bottom).

Next, we listed possible marker genes for each cluster (Supplementary Table S4). The lists of cluster 2 and cluster 3 markers included more than ten genes that had been identified as positive targets of Hh signaling in the previous study and are expressed in the peripheral region of the germ disc. Conversely, the list of cluster 5 markers included six genes that had been identified as negative targets of Hh signaling and are expressed in the central region. The cluster 4 markers included eight genes that had been shown to require negative regulation or both positive and negative regulations by Hh signaling and are expressed in the broad central or intermediate region. Some of the possible marker genes were cloned to show their specific expression in predicted regions of the germ disc (Figure 2D). These results suggested that single-cell transcriptome analysis, even when using a small number of early spider embryos, achieves sufficient resolution for separating 3 cell populations corresponding to the germ layers and successfully reconstructs the peripheral-to-central polarity (i.e., the future A-P polarity) in the ectodermal cell population.

### Global A-P Polarity of the Germ Disc

**FIGURE 3:**
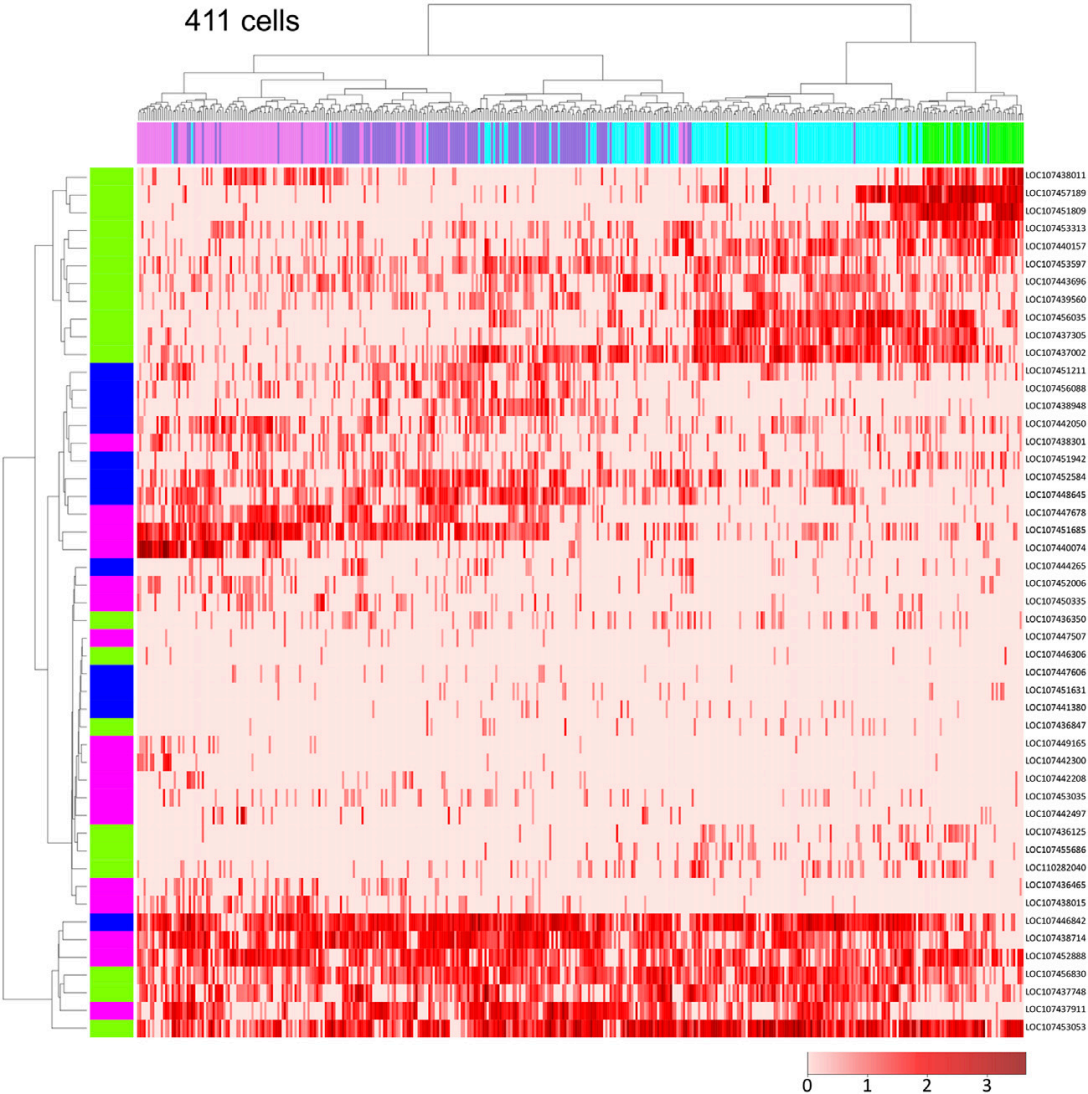
Single-cell transcriptome data showing global polarization of presumptive ectodermal cells. Dendrograms of 411 presumptive ectodermal cells (top) and *Pt-hh* and 48 genes regulated by Hh signaling (left) were constructed by hierarchical clustering of the normalized expression level of each gene in each cell. The bar on the top displays the cluster of the cells shown in Figure 2A: green, cluster 2; light blue, cluster 3; purple, cluster 4; magenta, cluster 5. The bar on the left shows the expression domain of genes in the germ disc revealed by WISH: green, peripheral region; blue, intermediate region; pink, central region. The heatmap shows the normalized expression levels of genes in the cells (The color code in the bottom right).

Next, we focused on the ectodermal cell population to investigate the diversity of cell states in detail. Using single-cell RNA-seq data on *Pt-hh* and 48 genes that are expressed in a specific region of the germ disc under the control of Hh signaling (Akiyama-Oda and Oda, 2020), we performed hierarchical clustering analysis of the 411 germ-disc cells belonging to the clusters 2-5. This analysis showed that the cells were roughly grouped in accordance with the clusters; however, cells from the neighboring clusters were not clearly separated but intermingled in the dendrogram (Figure 3). It was also shown that individual cell states were highly varied, but not completely different, exhibiting overlapping combinations of expressed genes. These results may represent the nature of the Hh signaling patterning system, which is able to produce a “fuzzy” French-flag-like pattern in a large cellular field. This nature of the Hh signaling patterning system revealed in the *Parasteatoda* embryo sharply contrasts with that of the maternal morphogen system in the *Drosphila* embryo, in which sharp boundaries of gap gene expression domains are established (Jaeger, 2011).

### Reconstruction of Mesodermal Cell Internalization From the Periphery of the Germ Disc

To investigate the separation of the germ layers, we focused on the mesodermal cell population, which internalizes from the periphery of the germ disc (Oda et al., 2007). This cell internalization starts at late stage 5, following the internalization of endodermal cells. We examined the transcript profiles of 140 cells belonging to the clusters 0–2 (Figure 2A), possibly corresponding to the presumptive endodermal and mesodermal cell populations and cells at the peripheral region of the germ disc, respectively, using 137 marker genes recognized in previous and ongoing studies. Hierarchical clustering analysis clearly separated the endodermal cells (cluster 0) from the mesodermal and ectodermal cells (clusters 1 and 2) with restricted expression of the cluster 0 markers (Supplementary Figure S3). Some of the cluster 1 markers were expressed at relatively high levels in both the mesodermal and endodermal cells. However, we did not detect cells that were in an intermediate state of the endoderm and mesoderm or the endoderm and ectoderm (Supplementary Figures. S2, S3). This result provided substantial evidence to suggest that “endodermal” cells were already in distinct cell states at late stage 5.

**FIGURE 4:**
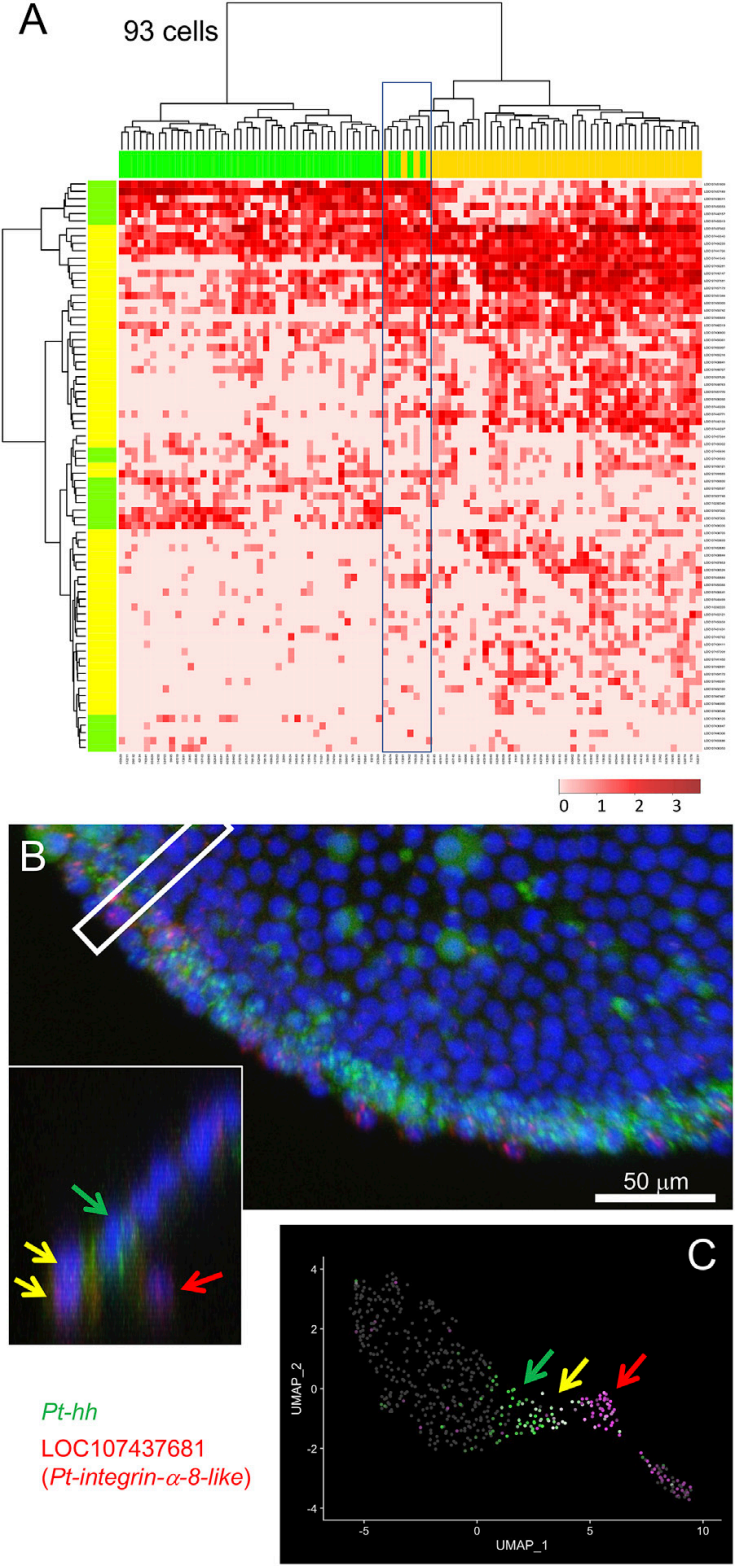
Specification of mesodermal cells at the peripheral region. **(A)** Dendrograms of 93 presumptive peripheral ectodermal and mesodermal cells (top) and 77 peripheral and mesodermal marker genes (left) were constructed by hierarchical clustering of the normalized expression level. The bar on the top displays the cluster of the cells shown in Figure 2A: yellow, cluster 1 (mesoderm); green, cluster 2 (peripheral ectoderm). The bar on the left shows the group of genes: yellow, mesodermal markers; green, peripheral markers which are the same genes shown in green in Figure 3. The heatmap shows the normalized expression levels (color code in the bottom right). Boxed cells show relatively high-level expression of both mesodermal and peripheral markers. **(B)** Detection of *Pt-hh* (green) and LOC107437681 (*Pt-integrin alpha-8-like*, red) transcripts using FISH at the peripheral region of the germ disc at late stage 5. The cross section of the boxed region is shown in the inset. Scale Bar = 50 μm. **(C)** Expression of the two genes visualized on the UMAP plot. In B and C, green arrows show *Pt-hh-*positive cells, red arrows show LOC107437681-positive cells, and yellow allows show double-positive cells.

Following exclusion of the endodermal cells, we examined the remaining 93 cells, possibly including mesodermal cells and cells at the peripheral region of the germ disc, using 77 selected marker genes. Hierarchical clustering analysis revealed the presence of two large cell populations in accordance with clusters 1 and 2, but with a small cell subpopulation displaying intermediate states (Figure 4A). To confirm whether cells displaying such intermediate states were present in the late stage-5 embryo, expression of a mesodermal (LOC107437681, *Pt-integrin alpha-8-like*) and a peripheral (LOC107451809, *Pt-hh*) gene was examined using FISH. As a result, cells that expressed both genes were observed at the edge of the germ disc (Figure 4B, yellow arrows), with adjacent cells on the germ-disc side expressing only the peripheral gene (Figure 4B, the green arrow) and nearby internal cells expressing only the mesodermal gene (Figure 4B, the red arrow). These observations suggested the possibility that the double-positive cells at the edge of the germ disc might be in an intermediate state undergoing commitment toward the mesodermal fate. The corresponding intermediate cell state was recognized in the UMAP plot (Figure 4C). These examples of data from single-cell RNA-seq analysis highlight the potential of this technique to follow cell state transitions in the developing spider embryo.

### Search for CM Cell Markers

**FIGURE 5:**
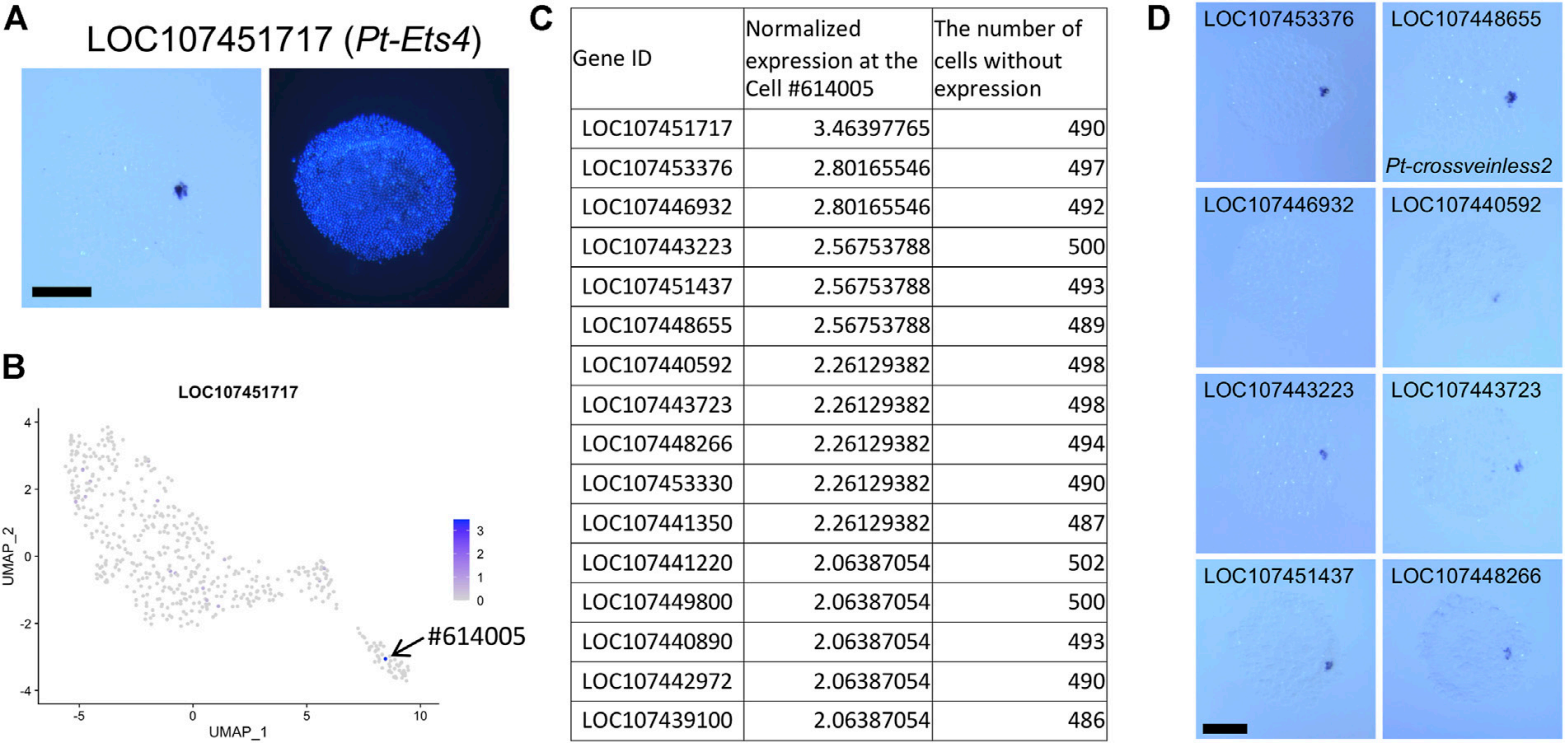
Identification of CM cell transcriptome. **(A)** Expression of LOC107451717 (*Pt-Ets4*) in a germ disc at late stage 5. The signal is detected at the CM cells. The right panel shows DAPI staining of the same embryo. **(B)** Expression of LOC107451717 visualized on the UMAP plot. Strong expression is detected specifically at the cell #614005 in the endodermal cluster. **(C)** The list of genes that show specific expression in the cell #614005. The normalized expression level in the cell #614005 and the number of cells (out of 505 cells) with no expression of the gene are shown. **(D)** Detection of transcripts of the top 9 genes (except LOC107451717) in the list. Signals are detected at the CM cells. Scale Bars = 200 μm.

The D-V axis of the spider embryo is specified by symmetry-breaking migration of clustered cells sending Dpp signals from the center of the germ disc following internalization (Akiyama-Oda and Oda, 2003, Akiyama-Oda and Oda, 2006, Akiyama-Oda and Oda, 2010). These cells are referred to as the CM cells. Known markers for this cell type include *Pt-dpp* (LOC107442925), *Pt-fascin (singed)* (At_eW_022_P10, LOC107440147), and *Pt-ets4* (LOC107451717) (Pechmann et al., 2017) (Figure 5A). To examine the comprehensiveness and practical use of our single-cell transcriptome data, we searched for additional CM-cell marker genes. Highly specific expression of *Pt-ets4* at the CM cells allowed us to identify a single cell #614005 demonstrating this cell type. This cell was grouped together with many other cells assigned to the presumptive endoderm (Figure 5B), strongly suggesting that the CM cells are part of the endoderm, not the mesoderm. Next, we listed up genes showing high levels of expression in the cell #614005 but no expression in most other cells (Figure 5C; Supplementary Table S5). The top-ranked gene in the list was *Pt-ets4* itself. Eight genes following *Pt-ets4* in the list were confirmed to be specifically expressed in the CM cells using WISH staining (Figure 5D). These results indicated that single-cell transcriptome data provide comprehensive resources that help to detect specific cell populations, even a small one, and marker genes for such cell types.

### Search for Genes With Similar Expression Pattern by Template Matching

**FIGURE 6:**
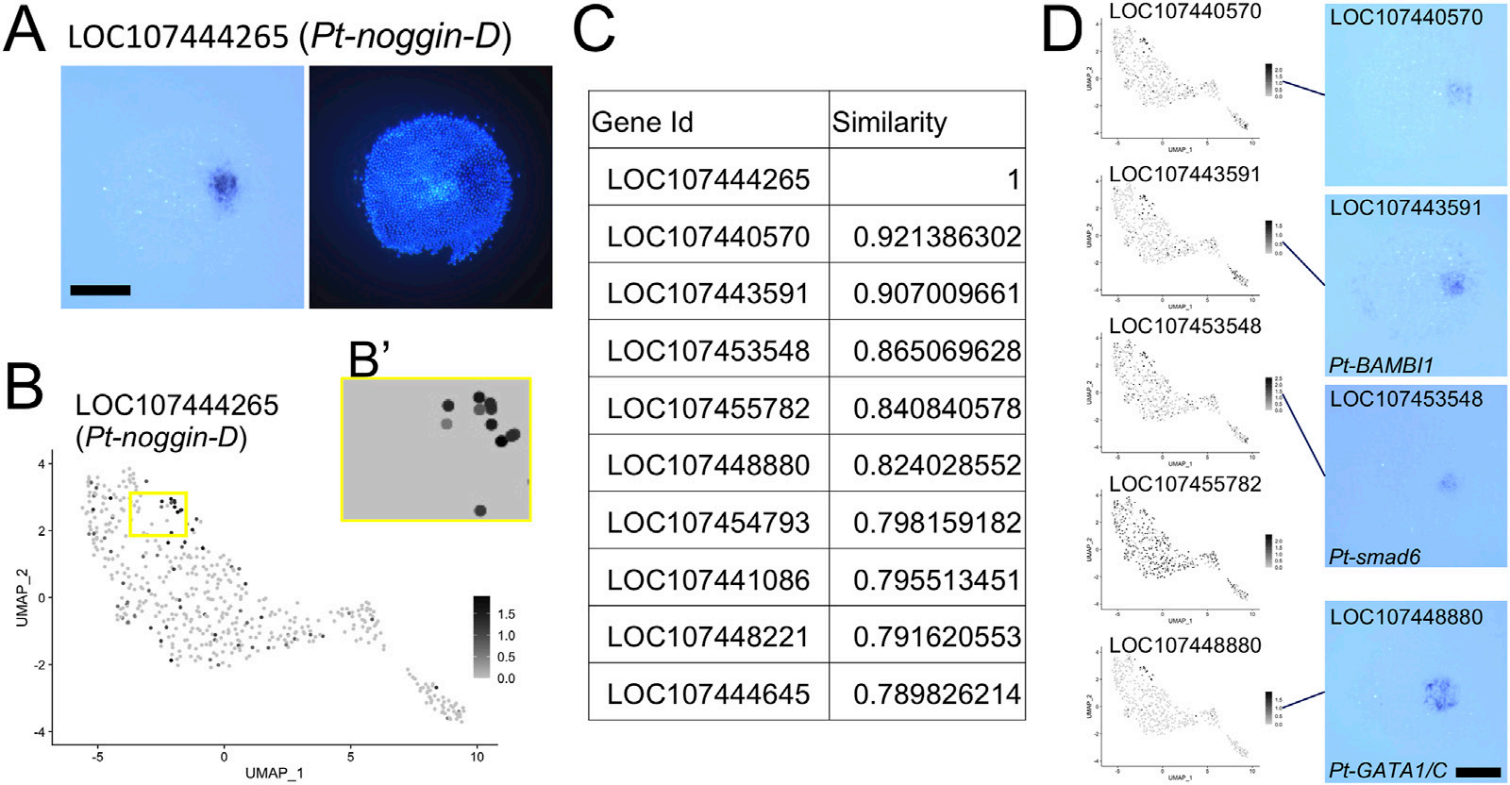
Identification of the cumulus on the UMAP plot. **(A)** Expression of LOC107444265 (*Pt-noggin-D*) in a germ disc at late stage 5. The signal is detected at the cumulus. The right panel shows DAPI staining of the same embryo. **(B)** Expression of LOC107444265 visualized on the UMAP plot. The region boxed in yellow was used as a template in the Open CV similarity search after the background color was changed to the color of cells without expression as in **(B′)**. **(C)** The list of genes with highest similarity scores in the similarity search. **(D)** Expression of the top 6 genes except LOC107444265 visualized on the UMAP plot and detected using WISH. *In situ* hybridization of LOC107455782 was not performed. Scale Bars = 200 μm.

We further attempted a gene search based on expression pattern similarity using the Open CV template matching module, which was applied to the UMAP plots displaying expression of individual genes. We focused on the region called the cumulus (Akiyama-Oda and Oda, 2003, Akiyama-Oda and Oda, 2006). Expression of several known genes, including LOC107444265 (*Pt-noggin-D*) (Figure 6A) and LOC107448880 (*Pt-gata1/gataC*) (Akiyama-Oda and Oda, 2010, Akiyama-Oda and Oda, 2020), is induced at the cumulus in response to the signals from the CM cells. As a simple example, we chose a rectangular region on the UMAP plot where several presumptive ectodermal (germ-disc) cells expressing high levels of *Pt-noggin-D* expression, which presumably correspond to the cumulus, are included together with several nonresponding cells (Figure 6B,B’). Using the rectangular region, we performed the template matching search against the corresponding regions of the UMAP plot images for all genes. Ten genes that were hit with highest similarity scores were listed, which included *Pt-noggin-D* itself and *Pt-gata1* (Figure 6C; Supplementary Table S6). The top 6 genes, except the fifth gene LOC107455782, whose expression was rather ubiquitous in the UMAP plot, were confirmed to show highly specific expression to the cumulus cells (Figure 6D). Application of a template matching tool to single-cell RNA-seq datasets provides a way to genome-widely identify genes whose expression is spatially regulated.

### Application of Single-Nucleus RNA-Seq for the Spider Embryo

Single-nucleus RNA-seq can be used to access mRNA (de novo transcripts or pre-mRNA) in nuclei. This technique is sometimes substituted for the single-cell RNA-seq technique, especially in cases where dissociation and collection of cells are difficult and target tissues are composed of divergent cell types (Grindberg et al., 2013; Habib et al., 2016; Zeng et al., 2016; Hu et al., 2017). We simultaneously applied single-cell and single-nucleus RNA-seq to the *Parasteatoda* germ-disc stage embryo to compare the performance of the two transcriptome techniques. In this second analysis, nuclei were extracted from 20 sibling embryos, and cells were subsequently dissociated from the same number of sibling embryos. Since the nuclei extraction took approximately 1 h 20 min to complete, the developmental stage for the nuclei sample was correspondingly earlier than those for the cell sample. When comparing the sampling timing of the cell samples between the first and second analyses, samples used for the second analysis were a little older (1–2 h) than those used for the first analysis. During the course of data analysis, the reference genome assembly of *Parasteatoda* was updated (Ptep_3.0). Therefore, we aligned RNA-seq reads against both the version-2 and version-3 genomes and also against the version-3 genome whose annotation was modified to count both intronic and exonic reads. As summarized in Supplementary Table S7, alignment against the version-3 genome resulted in detection of a slightly higher number of molecules than that against the version-2 genome in both single-cell and single-nucleus experiments. An increased number of molecules were detected when using the modified version-3 genome to analyze single-nucleus data. We used single-cell data aligned to the version-3 genome and single-nucleus data aligned to the modified version-3 genome for downstream analysis.

We followed similar procedures as the first single-cell data analysis (Figure 1B). After filtering out low-quality cells (Figure 1C), we performed a clustering analysis. In the single-cell analysis, germ-disc epithelial cells (presumptive ectodermal cells) with polarized expression of A-P marker genes were separated into two large clusters similar to the first single-cell analysis (Supplementary Figures S1, S4A), although this time the two clusters did not differ in the numbers of genes and molecules detected per cell. The situation of the single-nucleus analysis result was distinct; in the UMAP plot, presumptive ectodermal cells were distributed in one large mass with mesodermal cells, and the first split of ectodermal subclusters was detected between the peripheral cells and others (Supplementary Figure S4B).

The following analyses were performed with data that excluded 85 genes identified as DEGs of the two ectodermal clusters in this single-cell analysis (Supplementary Table S8), which showed ubiquitous and high levels of expression. The clustering analysis using this dataset showed similar results as in the first single-cell analysis (Supplementary Figure S5; Figure 2). We detected three germ layers and the A-P polarization of germ-disc cells in the single-nucleus as well as the single-cell analysis. The CM cells and a cluster for the cumulus cells were also detected. However, there was one uncharacterized cluster in the single-cell analysis (cluster 5 in Supplementary Figure S5A) and one cluster consisting of nuclei with relatively low numbers of genes and molecules in the single-nucleus analysis (cluster 7 in Supplementary Figure S5B). These indicated that the single-nucleus and single-cell RNA-seq analyses produced mostly consistent results, but one difference between the two methods was found: single-cell RNA-seq, unlike single-nucleus RNA-seq, was affected by the expression levels of a set of genes expressed ubiquitously and at high levels.

### Identification of Dynamic Cell States by Single-Nucleus Transcriptome

**FIGURE 7:**
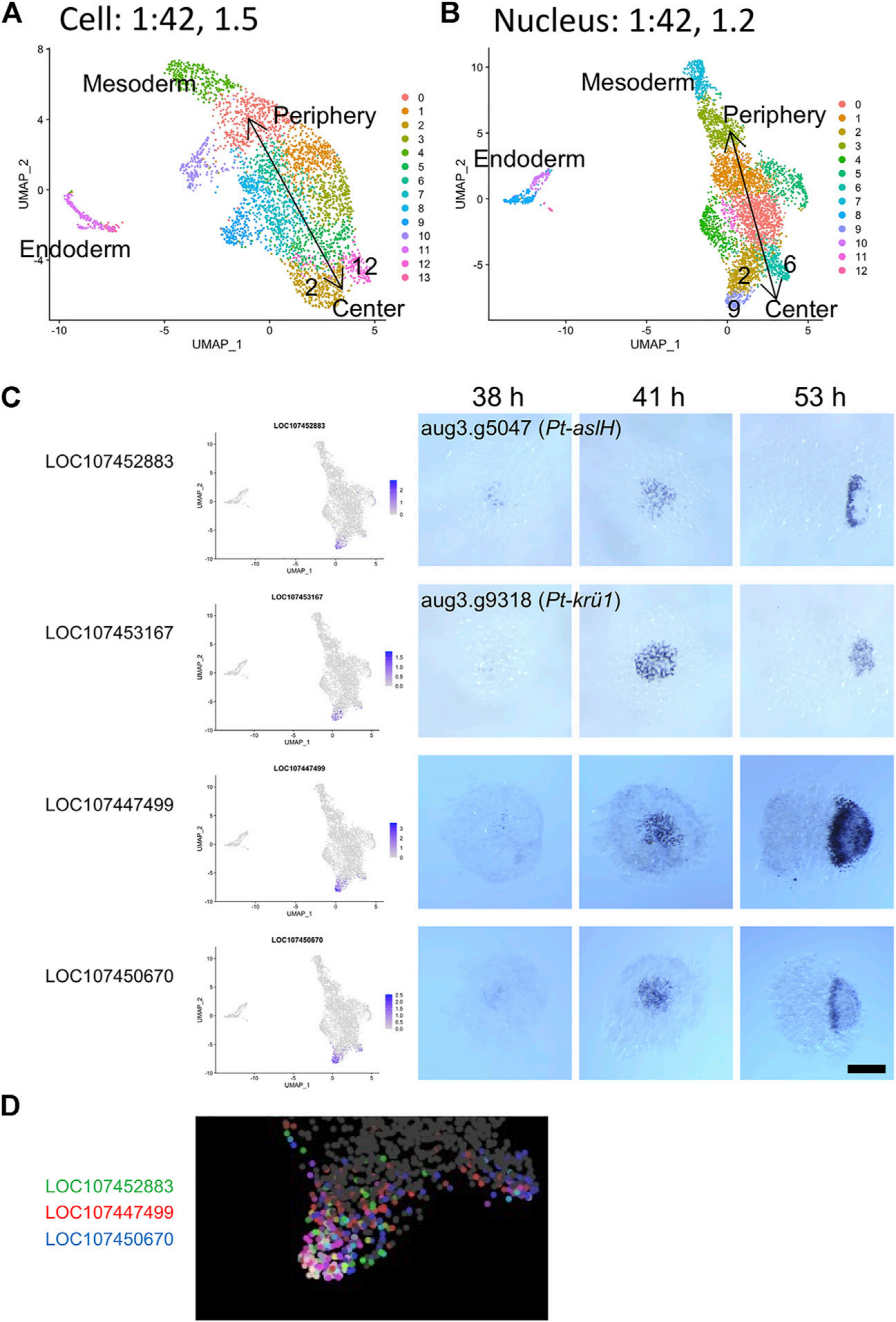
Detection of a dynamically emerging cell state using single-nucleus transcriptomics. **(A,B)** The UMAP plots of 3,621 cells **(A)** and 4,720 nuclei **(B)**, showing clusters generated from the second single-cell **(A)** and the single-nucleus **(B)** transcriptome data. The clustering was performed using 42 PCs and with resolution parameters of 1.5 **(A)** and 1.2 **(B)**. Annotations of the germ layers and the reconstructed orientation of the germ disc are indicated. **(C)** Expression of cluster 9 marker genes identified in the single-nucleus transcriptome analysis. The expression is visualized on the UMAP plot (left) and is detected using WISH. The stained embryos are at late germ-disc stage (38 h, late stage 5), the start of germ-disc to germ-band transition stage (41 h, early stage 6), and early germ-band stage (53 h, stage 7). Embryos shown in the right panels (53 h) are oriented anterior to the left. Scale Bar = 200 μm. The images of embryos stained for *Pt-aslH* and *Pt-krü1* transcripts are from Akiyama-Oda and Oda (2020) (CCBY4.0). **(D)** Expression of three cluster 9 marker genes visualized in three colors on the UMAP plot. The cluster 9 region is enlarged.

Next, to compare the capabilities of detecting and separating dynamic cell states in the single-cell and single-nucleus transcriptome analyses, we tested higher resolution parameters. As shown in Supplementary Figure S5, the clustering analysis at a low-resolution parameter identified four clusters of ectodermal cells corresponding to peripheral-to-central (future anterior-to-posterior) series of regions of the germ disc both in the single-cell (clusters 3, 0, 1, 2 in Supplementary Figure S5A) and single-nucleus analysis (clusters 3, 2, 1, 0 in Supplementary Figure S5B). Using higher resolution parameters, more clusters were identified (Figure 7A). Here, we focused on two types of dynamic gene expression that are known to occur in the central region of the germ disc. One of the two originates from the progressive activation of Delta-Notch signaling from around the center of the germ disc, which leads to the formation of a salt-and-pepper pattern of gene expression that reflects the specification of the caudal mesodermal and caudal ectodermal cell fates (Oda et al., 2007). The other is initiation of oscillatory gene expression that contributes to the formation of segmental stripes in the posterior ectoderm (Akiyama-Oda and Oda, 2020). In the single-cell analysis using a high-resolution parameter, the central cluster (cluster 2 in Supplementary Figure S5A) was split into two subclusters (clusters 2 and 12 in Figure 7A), while in the single-nucleus analysis, cluster 0 in Supplementary Figure S5B was split into three subclusters (clusters 2, 6, and 9 in Figure 7B). Both the cluster 2s were comparable to each other, representing cell states in the wide central region similar to those detected with the low-resolution parameters. Cluster 12 in the single-cell analysis was comparable to cluster 6 in the single-nucleus analysis. Both clusters were characterized by expression of *Pt-Delta* and two other genes (LOC107440074, LOC107442300; Supplementary Table S9; Supplementary Figure S6), representing the cell state specified as the caudal mesoderm. The additional cluster 9 in the single-nucleus analysis was characterized by expression of *Pt-aslH*, *Pt-krü1*, and other genes (LOC107447499, LOC107450670), which were faintly detected in cells around the center of the germ disc at late stage 5 (38 h AEL) and strongly detected in a broader area at early stage 6 (41 h AEL) (Figure 7C). The expression of these genes was developed to exhibit a dynamic pattern which varied depending on the gene in the opisthosomal region (53 h AEL). The location of the cluster 9 on the UMAP plot in the single-nucleus analysis was in accordance with the reconstructed global polarity in the ectodermal cell population, with the emerging caudal mesodermal cell population (cluster 6) being separated. Moreover, differential ranges of the expression regions of the cluster 9-specific genes were recognized on the UMAP plot (Figure 7D), possibly capturing ordered transcriptional activations in the posterior terminal cell population. In contrast to the single-nucleus analysis, the single-cell analysis did not detect the corresponding posterior terminal cell population as a separate subcluster even when parameters for the dimension and resolution were raised. Nevertheless, cell states characterized by expression of the four genes (*Pt-aslH*, *Pt-krü1*, LOC107447499, and LOC107450670) were found in a limited area in the posterior terminal cell population on the UMAP plot, but the polarity reconstruction was imperfect (Supplementary Figure S7). Considering that the sampling timing for the single-cell transcriptome was a little later than that for the single-nucleus transcriptome, the poorer resolution of the single-cell transcriptome was likely due to lower sensitivity to the emerging cell states. Collectively, these results suggested that single-nucleus RNA-seq is more powerful than single-cell RNA-seq in detecting dynamic cell states in early spider embryos.

### Future Applications of Single-Cell and Single-Nucleus Transcriptome Techniques in the Spider Model System

In this study, we showed that the singe-cell/nucleus transcriptome techniques are applicable to examination of patterning of early spider embryos. Our results indicate that these techniques exhibit different features. Owing to a higher sensitivity, the single-nucleus RNA-seq enables detection of dynamic cell states. On the other hand, because the single-cell RNA-seq can detect mRNA molecules in the cytoplasm, data obtained with this technique are suitable for quantitative comparison with gene expression data obtained by FISH and can be used to spatial reconstruction of gene expression.

There are many merits of using singe-cell/nucleus transcriptome techniques for studying the patterning processes in *Parasteatoda*. Most previous studies using this spider model system have relied on molecular and genetic knowledge accumulated in the well-studied model organisms, such as *Drosophila*. Application of these techniques, however, allows us to focus our research on components of the *Parasteatoda* genome in an unbiased way. The fact that the gene expression patterns in the early spider embryo are reconstructed to a large extent by the single-nucleus transcriptome indicates that genome-wide discovery of genes whose expression is spatially regulated is possible without laborious staining of embryos. Moreover, if such gene discovery is combined with functional gene screening (e.g., parental and embryonic RNAi), as done in some previous studies (Oda et al., 2007; Akiyama-Oda and Oda, 2010, Akiyama-Oda and Oda, 2020; Kanayama et al., 2011; Pechman et al., 2017), we would be able to identify key genes necessary for specific steps in the patterning processes.

When the sequenced genome is available for a species, a popular method to find genes of interest is to search for sequences using sequence similarity detection tools, such as BLAST. Given that the gene expression patterns are reconstructed, an alternative can be to search patterns using image similarity detection tools, such as template matching. However, we will require a web-based application to facilitate this type of gene searches.

In this work, our analyses were focused on the late stage 5 of *Parasteatoda* embryonic development. An issue that should be addressed is to what extent the gene expression patterns can be reconstructed by single-cell/nucleus transcriptome analyses when using older embryos, in which more complicated patterns of gene expression have been developed. If pattern reconstruction is achieved in a broad range of developmental stages, we might be able to analyze time-series samples to obtain clues about genetic components that contribute to driving pattern-forming processes.

## Conclusion

This work has demonstrated that single-cell and single-nucleus RNA-seq analyses are applicable to the germ-disc stage embryo in *Parasteatoda*. Both techniques worked effectively using only twenty or fewer embryos as the starting material for each experiment. UMAP clustering reveals separation of the three germ layers and a reconstruction of the global polarity in the presumptive ectodermal cell population. Additionally, we showed that the data resources generated by the transcriptome analyses help to conduct a genome-wide search for genes whose expression is spatially regulated, based on the detection of pattern similarity. We also showed that single-nucleus transcriptome analysis is more powerful than single-cell transcriptome analysis in detecting dynamic cell states in developing spider embryos. We propose that future applications of these transcriptome techniques to *Parasteatoda* embryos at broader developmental stages may help to study the pattern formation in a simple cellular field, which cannot be accessible by the *Drosophila* blastoderm embryo.

## Data Availability

The datasets presented in this study can be found in online repositories. The names of the repository/repositories and accession number(s) can be found at: GEO accession GSE201705.
