# Supplementary material for: Reconstruction of the Global Polarity of an Early Spider Embryo by Single-Cell and Single-Nucleus Transcriptome Analysis
Source: Front Cell Dev Biol. 2022 Jul 22;10:933220. doi: 10.3389/fcell.2022.933220 (PMC9353575; doi:10.3389/fcell.2022.933220)
Supplement: Supplementary file 5 [file DataSheet1.pdf]

Supplementary Material for

Reconstruction of the Global Polarity of an Early Spider Embryo by Single-Cell and Single-Nucleus Transcriptome Analysis

Yasuko Akiyama-Oda[1,2,3], Takanori Akaiwa[1,4], and Hiroki Oda[1,4]

1. JT Biohistory Research Hall, Takatsuki, Japan; 2. PRESTO, Japan Science and Technology Agency, Kawaguchi, Japan; 3. Department of Microbiology and Infection Control, Faculty of Medicine, Osaka Medical and Pharmaceutical University, Takatsuki, Japan; 4. Department of Biological Science, Graduate School of Science Osaka University, Toyonaka, Japan.

Captions for Supplementary Figures:

Supplementary Figure 1 | Clustering analysis of the single-cell transcriptomes.
Supplementary Figure 2 | Expression of marker genes of the endodermal and mesodermal clusters.
Supplementary Figure 3 | Characterization of mesodermal and endodermal cells.
Supplementary Figure 4 | Clustering analysis of the second single-cell and the single-nucleus transcriptomes.
Supplementary Figure 5 | Detection of germ layers and reconstruction of ectodermal polarity by single-cell and single-nucleus transcriptome analysis.
Supplementary Figure 6 | Expression of marker genes of a central subcluster.
Supplementary Figure 7 | Expression of genes showing dynamic expression in the central region of the germ disc.

Captions for Supplementary Tables:

Supplementary Table 1 | Statistics of the first single-cell RNA-seq experiment.
Supplementary Table 2 | 445 DEGs between two presumptive ectodermal clusters.
Supplementary Table 3 | Cell cycle genes.
Supplementary Table 4 | Cluster Markers identified in the first single-cell RNA-seq analysis.
Supplementary Table 5 | Genes expressed specifically in the CM cells.
Supplementary Table 6 | Genes with highly similar pattern to LOC107444265 cumulus expression pattern on the UMAP plot.
Supplementary Table 7 | Statistics of the second single-cell and the single-nucleus RNA-seq experiment.
Supplementary Table 8 | 85 DEGs between two presumptive ectodermal clusters.
Supplementary Table 9 | Markers of clusters 6 and 9 identified in the single-nucleus RNA-seq analysis.
Supplementary Table 10 | Clones.

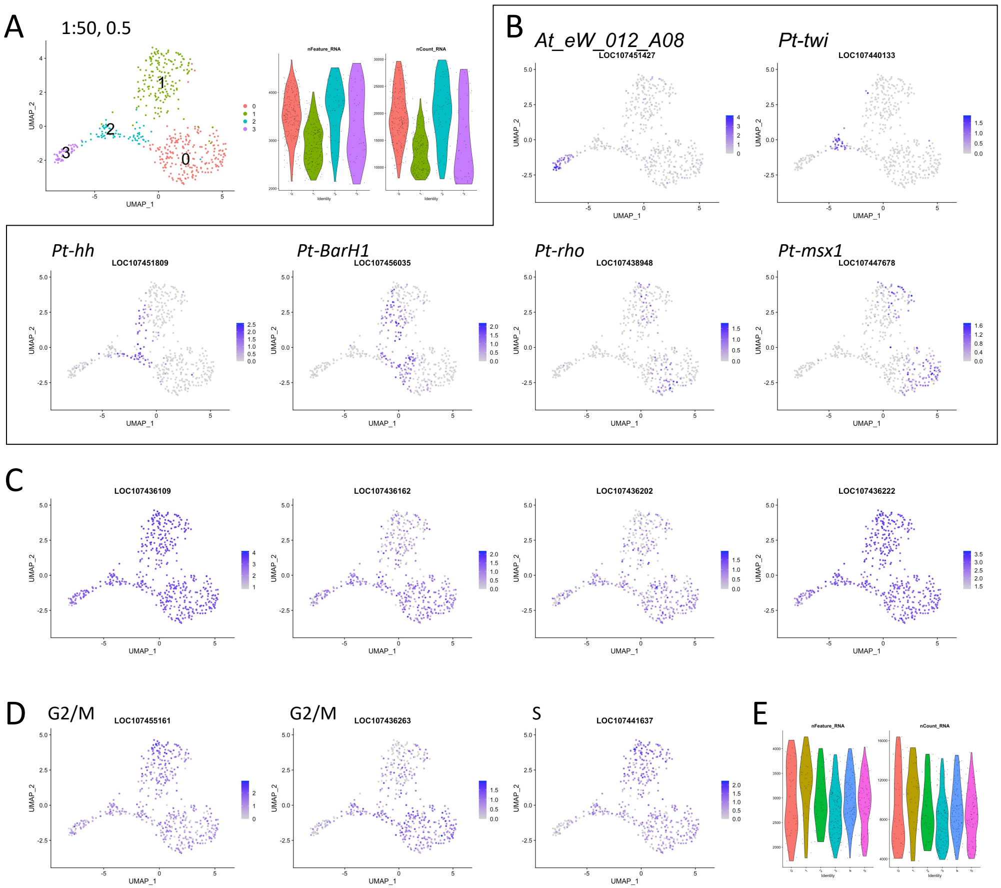

Supplementary Figure 1 | Clustering analysis of the single-cell transcriptomes. (A) The UMAP plot of 505 late germ-disc cells, showing presumptive ectodermal (clusters 0, 1), mesodermal (cluster 2), and endodermal (clusters 3) clusters. The cells were clustered using 50 PCs with a resolution parameter of 0.5. Violin plots show the number of genes (nFeature_RNA) and RNA molecules (nCount_RNA) detected in cells of each cluster. Among the two clusters of presumptive ectodermal cells, cluster 1 consists of cells with relatively small numbers of molecules and genes. The 445 DEGs identified between clusters 0 and 1 (Table S2) were excluded in the analysis shown in Figs. 2-6, S1E, S2, and S3. (B) Expression of germ-layer marker genes and AP marker genes visualized on the UMAP plot with the color code of the normalized expression levels. *At_eW_012_A08*, endoderm; *Pt-twi* (*twist*), mesoderm; *Pt-hh* (*hedgehog*), peripheral region; *Pt-BarH1*, broad peripheral region; *Pt-rho* (*rhomboid*), intermediate region; *Pt-msx1*, central region. The peripheral-to-central polarity is observed in both the presumptive ectodermal clusters. (C) Expression of four of the DEGs identified between the clusters 0 and 1 (Table S2). Their expression is detected ubiquitously, although expression levels differ between the two ectodermal clusters. (D) Expression of three of the cell-cycle genes (Table S3). LOC107436263 was also identified as a DEG between the clusters 0 and 1. (E) Violin plots of cells in each cluster shown in Fig. 2A.

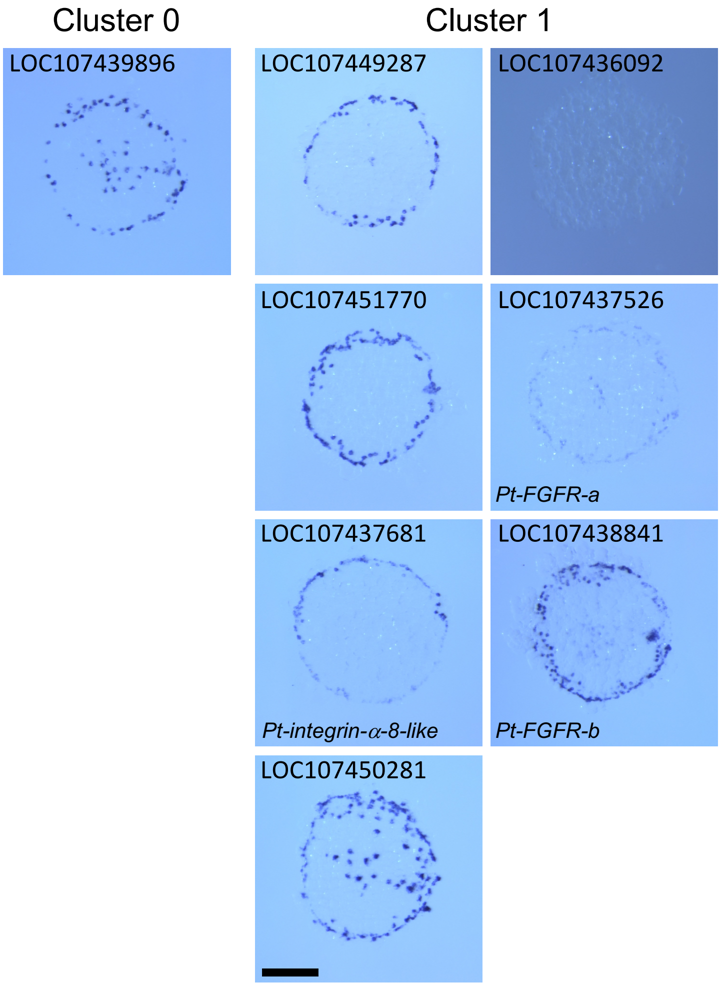

Supplementary Figure 2 | Expression of marker genes of the endodermal and mesodermal clusters. The cluster markers were identified in the analysis shown in Fig. 2A. Cluster 0, endoderm; cluster 1, mesoderm. LOC107450281 is expressed in both the mesoderm and endoderm. The embryos are at the late germ-disc stage (late stage 5). Scale Bar = 200 µm.

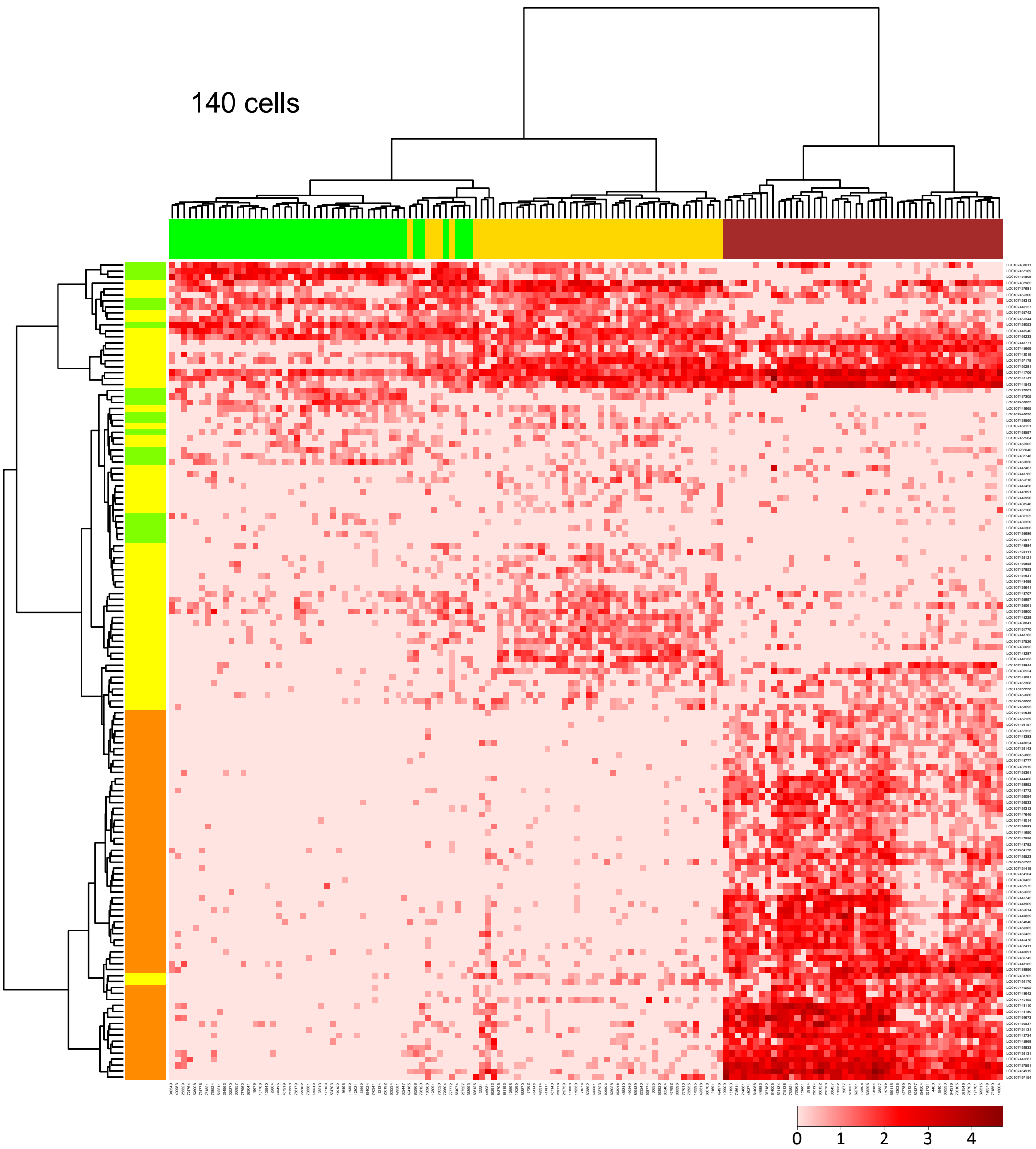

Supplementary Figure 3 | Characterization of mesodermal and endodermal cells. Dendrograms of 140 presumptive peripheral ectodermal, mesodermal, and endodermal cells (top) and 137 marker genes of mesodermal, endodermal, and peripheral cells (left) were constructed by hierarchical clustering on the normalized expression level of each gene in each cell. The bar on the top displays the cluster of cells shown in Fig. 2A: brown, cluster 0 (endoderm); yellow, cluster 1 (mesoderm); green, cluster 2 (peripheral ectoderm). The bar on the left shows the group of genes: orange, endodermal markers; yellow, mesodermal markers; green, peripheral markers which are the same genes shown in green in Fig. 3. The heatmap shows the normalized expression levels of the genes in the cells (The color code present in the bottom right). The endodermal cells (brown) compose an independent cluster.

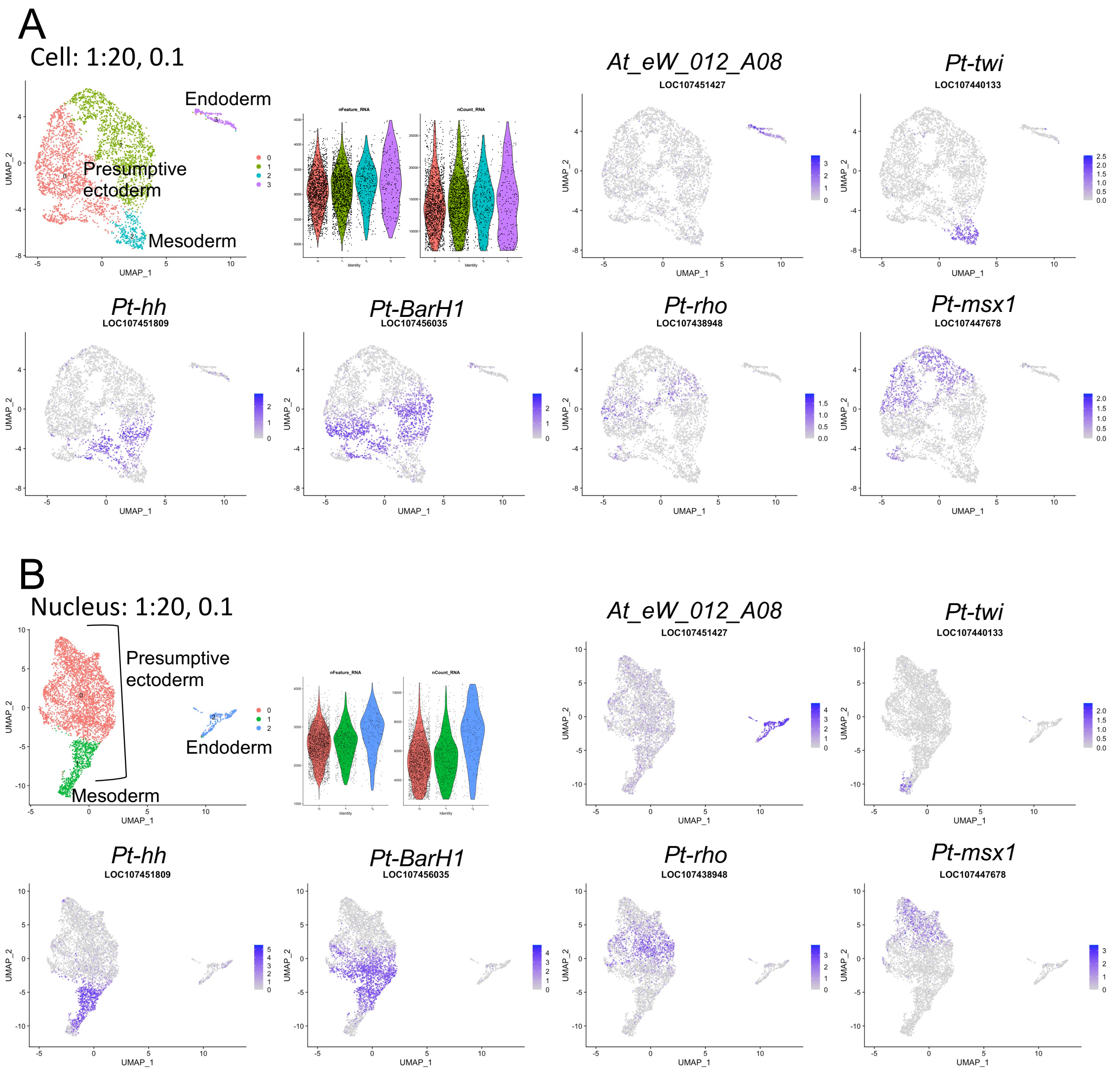

Supplementary Figure 4 | Clustering analysis of the second single-cell and the single-nucleus transcriptomes. (A, B) The UMAP plots of 3,621 cells (A) and 4,720 nuclei (B), showing presumptive ectodermal (clusters 0, 1), mesodermal (cluster 2), and endodermal (clusters 3) clusters in the single-cell analysis (A), and endodermal (cluster 2), mesodermal plus peripheral ectodermal (cluster 1), and other ectodermal (cluster 0) clusters in the single-nucleus analysis (B). Clustering was performed using 20 PCs and with a resolution parameter of 0.1 (A, B). Violin plots show the number of genes (nFeature_RNA) and RNA molecules (nCount_RNA) detected in cells (A) and nuclei (B) of each cluster. Expression of germ-layer marker genes and AP marker genes are visualized on the UMAP plot. *At_eW_012_A08*, endoderm; *Pt-twi* (*twist*), mesoderm; *Pt-hh* (*hedgehog*), peripheral region; *Pt-BarH1*, broad peripheral region; *Pt-rho* (*rhomboid*), intermediate region; *Pt-msx1*, central region. The peripheral-to-central polarity is observed in the two presumptive ectodermal clusters of the single-cell UMAP and in the single-nucleus UMAP. The 85 DEGs identified between the clusters 0 and 1 of this single-cell analysis (A) were excluded while performing the analysis shown in Fig. 7 and Figs. S5-S7.

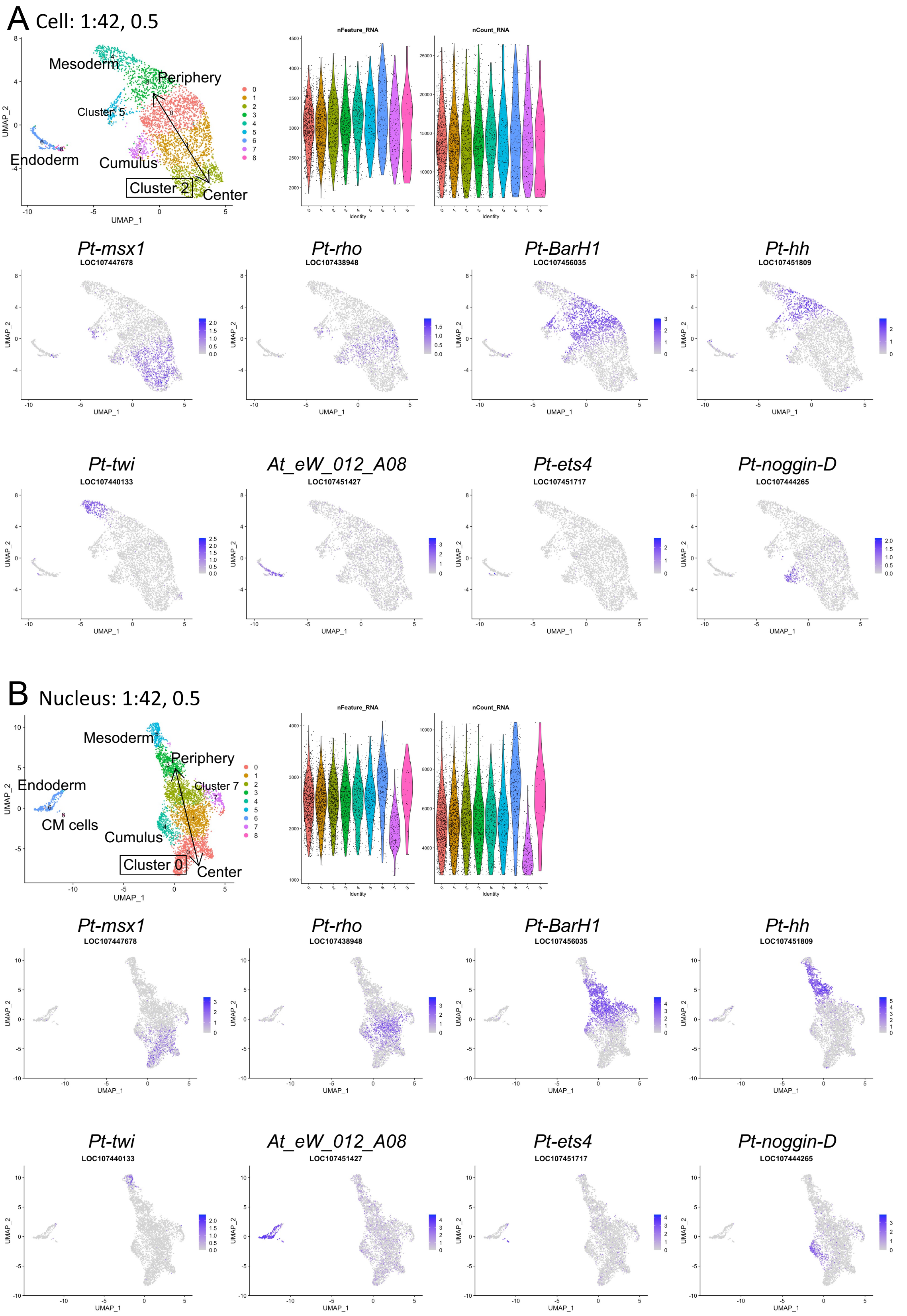

Supplementary Figure 5 | Detection of germ layers and reconstruction of ectodermal polarity by

single-cell and single-nucleus transcriptome analysis. (A, B) The UMAP plots showing results of the clustering analysis of the second single-cell (A) and the single-nucleus (B) transcriptomes using 42 PCs with a relatively lower resolution parameter of 0.5. Annotations of cell-types and the reconstructed orientation of the germ disc are indicated. Violin plots show the number of genes (nFeature_RNA) and RNA molecules (nCount_RNA) detected in cells (A) and nuclei (B) of each cluster. Expression of known marker genes on the UMAP plot. *Pt-msx1*, central region; *Pt-rho* (*rhomboid*), intermediate region; *Pt-BarH1*, broad peripheral region; *Pt-hh* (*hedgehog*), peripheral region; *At_eW_012_A08*, endoderm; *Pt-twi* (*twist*), mesoderm; *Pt-ets4*, CM cells; *Pt-noggin-D*, cumulus. The cluster 5 detected in the single-cell analysis is unannotated (A), and the cluster 7 detected in the single-nucleus analysis consists of cells with lower number of molecules and genes (B).

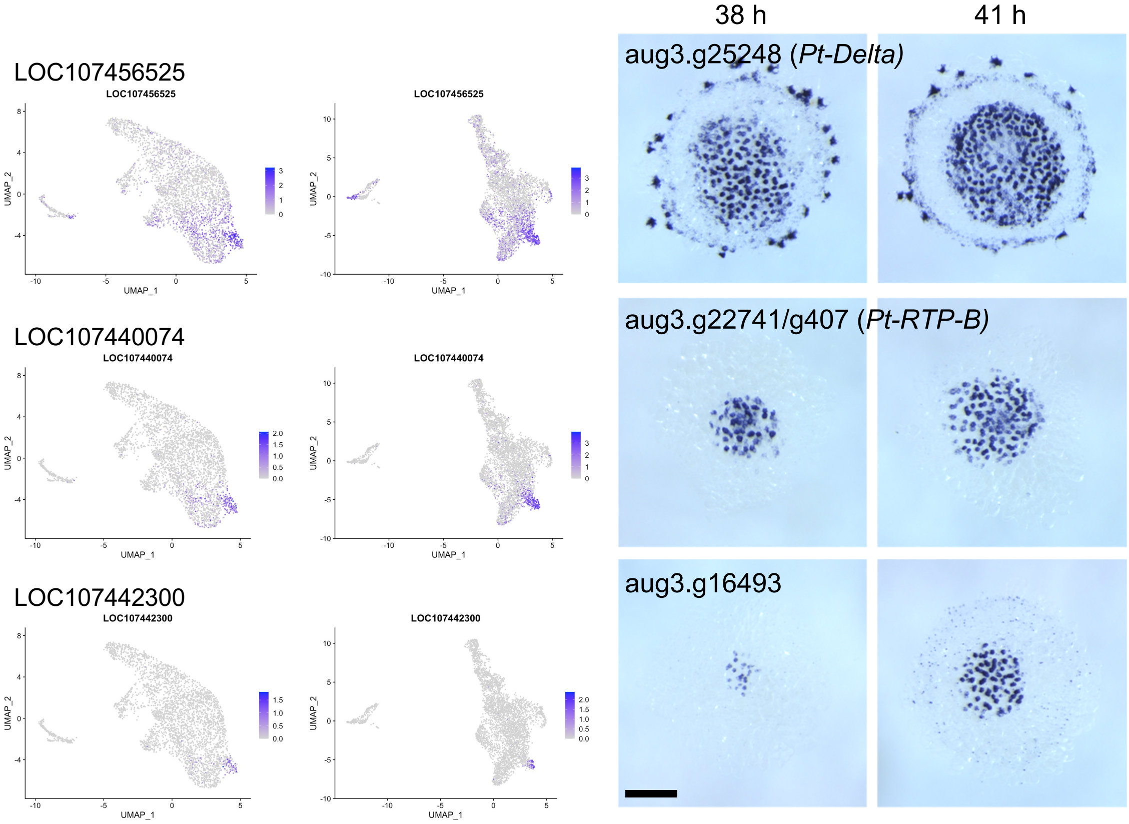

Supplementary Figure 6 | Expression of marker genes of a central subcluster. Expression of cluster 6 marker genes identified in the single-nucleus transcriptome analysis (Fig. 7B) are visualized on the UMAP plots (Fig. 7A, B) and using WISH. The stained embryos are at late germ-disc stage (38 h, late stage 5) and at the start of germ-disc to germ-band transition stage (41 h, early stage 6). Scale Bar = 200 µm. The images of the embryos are from Akiyama-Oda and Oda (2020) (CCBY4.0).

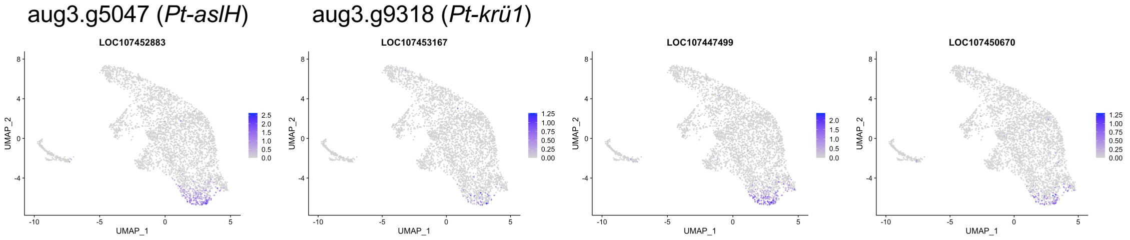

Supplementary Figure 7 | Expression of genes showing dynamic expression in the central region of the germ disc. Expression of cluster 9 marker genes identified in the single-nucleus transcriptome analysis (Fig. 7B) was visualized on the UMAP plot generated from the second single-cell transcriptome data (Fig. 7A). Their expression is detected in a similar region.
